# Salivary detection of SARS‐CoV‐2 (COVID‐19) and implications for oral health‐care providers

**DOI:** 10.1002/hed.26322

**Published:** 2020-06-13

**Authors:** Nimit Bajaj, Bruno P. Granwehr, Ehab Y. Hanna, Mark S. Chambers

**Affiliations:** ^1^ Department of Radiation Oncology The University of Texas MD Anderson Cancer Center Houston Texas USA; ^2^ Department of Infectious Diseases The University of Texas MD Anderson Cancer Center Houston Texas USA; ^3^ Department of Head and Neck Surgery The University of Texas MD Anderson Cancer Center Houston Texas USA

**Keywords:** COVID‐19, head and neck, nosocomial infection, saliva, SARS‐CoV‐2

## Abstract

The coronavirus disease 2019 (COVID‐19) pandemic has become a major public health crisis. The diagnostic and containment efforts for the disease have presented significant challenges for the global health‐care community. In this brief report, we provide perspective on the potential use of salivary specimens for detection and serial monitoring of severe acute respiratory syndrome coronavirus 2 (SARS‐CoV‐2), based on current literature. Oral health‐care providers are at an elevated risk of exposure to COVID‐19 due to their proximity to nasopharynx of patients, and the practice involving the use of aerosol‐generating equipment. Here, we summarize the general guidelines for oral health‐care specialists for prevention of nosocomial transmission of COVID‐19, and provide specific recommendations for clinical care management.

## 1 INTRODUCTION

An outbreak of pneumonia of unknown etiology was detected in Wuhan, Hubei Province of China, in late December, 2019.^1^, ^2^ Since then, the disease has rapidly spread around the globe. The causative agent of the disease was identified to be a novel coronavirus of bat origin,^3^ later termed severe acute respiratory syndrome coronavirus 2 (SARS‐CoV‐2),^4^ and the disease was named coronavirus disease 2019 (COVID‐19).^5^ The World Health Organization (WHO) designated the COVID‐19 outbreak as a pandemic on March 11, 2020.^6^ As of May 19, 2020, there have been over 4 731 458 laboratory confirmed cases and 316 169 deaths reported globally. In the United States alone, more than 1 477 516 COVID‐19 cases and 89 272 deaths have been reported, and the numbers continue to rise.^7^, ^8^

Many patients infected with SARS‐CoV‐2 are asymptomatic; however, the most common symptoms at the onset of illness are fever, cough, dyspnea, and myalgia.^9^, ^10^ Some patients may also experience headache, dizziness, loss of taste and/or smell,^11^ and gastrointestinal symptoms such as nausea, vomiting, and diarrhea.^10^, ^12^ Chest computed tomography (CT) findings of patients with COVID‐19 show multifocal bilateral ground‐glass opacities and areas of consolidation.^10^, ^13^ Severe‐onset disease may lead to acute respiratory distress syndrome and death.^12^ SARS‐CoV‐2 is thought to spread primarily through respiratory droplets and from close person‐to‐person contact with an infected individual.^14^ The virus has also been shown to survive on surfaces such as plastic and stainless steel for up to 72 hours.^15^ Currently, the recommended mode of diagnostic specimen collection is from the upper respiratory tract using nasopharyngeal and oropharyngeal swabs. However, this requires close contact between the health‐care worker and individual, and may induce sneezing and coughing which can lead to aerosol generation, and cause transmission of the virus. This method of sample collection may also cause discomfort and bleeding in some people.^16^ In addition, there is an acute shortage of swabs and protective gear, and an overburdening of the testing centers. Thus, there is a need to explore other evidence‐based modalities of specimen collection for mass testing and monitoring of COVID‐19.

## 2 DIAGNOSTIC POTENTIAL OF SALIVA FOR SARS‐COV‐2

It has been reported that the angiotensin converting enzyme II (ACE2) is the host cell receptor to which the SARS‐CoV‐2 binds to gain entry into cells, same as SARS‐CoV.^9^, ^17^ Xu et al have demonstrated that the receptor binding domain of SARS‐CoV‐2 spike protein supports strong interactions with the human ACE2 receptor.^18^ The ACE2 protein is present in most organs of the human body and is abundantly expressed in the vascular endothelial cells, heart, alveolar epithelial cells of lungs, and enterocytes of the intestine.^19^ These findings indicate that these organs may potentially be at high risk for COVID‐19 infection.^20^ Recently, RNA sequencing studies from the Cancer Genome Atlas database have identified that there is a high expression of the ACE2 receptors on the epithelial cells of oral mucosa.^21^ Among oral sites, the highest expression was seen in the epithelial cells of tongue, followed by buccal and gingival tissues. These findings may provide clues for further investigation of oral routes of infection, pathogenesis and detection of COVID‐19.

Previous studies have demonstrated that salivary specimens have a higher than 90% concordance rate with nasopharyngeal specimens in the detection of respiratory viruses.^22^ In an initial pilot study by To et al,^23^ SARS‐CoV‐2 was detected in the salivary specimens of 11 out of 12 patients with laboratory‐confirmed COVID‐19, and all 33 individuals who tested negative for nasopharyngeal specimens also tested negative for salivary specimens. In another recently published study, posterior oropharyngeal saliva samples were collected for 23 patients with laboratory‐confirmed COVID‐19 for nasopharyngeal specimens.^24^ Of these, 20 patients (87%) tested positive for SARS‐CoV‐2 in their saliva. Serial viral load was ascertained using reverse transcriptase quantitative polymerase chain reaction (RT‐qPCR). It was found that the salivary viral load was highest during the first week after symptom onset and it declined over time. The salivary sample was self‐collected by patients by coughing up saliva from the posterior oropharynx. Therefore it is possible that these specimens included secretions from the nasopharynx or the lower respiratory tract, rather than being completely salivary. In a study by Williams et al,^25^ 622 individuals were tested for COVID‐19 using nasopharyngeal specimens, of which 522 also provided salivary specimens. For salivary specimen collection, they were instructed to pool saliva in their mouth for 1 to 2 minutes, and gently spit into a collection pot. Thirty‐three of the 39 patients (84.6%) who tested positive for nasopharyngeal specimens also had SARS‐CoV‐2 detected in their saliva.^25^ A study of 44 inpatients with COVID‐19 noted a high correlation between nasopharyngeal and salivary samples, with higher viral titers in saliva.^26^ It was found that the salivary specimens yielded higher detection sensitivity and consistency throughout the course of disease. This study also enrolled 98 asymptomatic health‐care workers, who self‐collected nasopharyngeal and salivary specimens every 3 days for a period of 2 weeks. SARS‐CoV‐2 was detected in saliva from two health‐care workers who tested negative for matching nasopharyngeal samples.^26^ These data, although limited, suggest that saliva may be more sensitive in detection of asymptomatic or pre‐symptomatic infections.

Researchers from Rutgers University evaluated the use of salivary specimens for SARS‐CoV‐2 detection in symptomatic patients from three ambulatory care centers.^27^ They found 100% positive and negative concordance between results obtained from testing of saliva and those obtained from nasopharyngeal and oropharyngeal swabs [saliva vs nasopharyngeal swab: (26/26) positive agreement, (27/27) negative agreement; saliva vs oropharyngeal swab: (4/4) positive agreement, (3/3) negative agreement]. The US Food and Drug Administration (FDA) has since issued them an emergency use authorization for the use of salivary specimens, in addition to the nasopharyngeal and oropharyngeal swabs, for detection of SARS‐CoV‐2 RNA in individuals suspected of COVID‐19.^27^ A salivary diagnostic test would allow for a noninvasive self‐administered sample collection under health‐care providers' directive for individuals in quarantine, and would circumvent the issues regarding global shortage of swabs and personal protective equipment (PPE) needed for conventional COVID‐19 testing. Specific guidelines are needed to standardize the method for collection of salivary specimens, and implement the use of appropriate assays, and processing methods. The presence of SARS‐CoV‐2 in the saliva of infected patients also bears implications for a high potential of transmission in the dental operatory, and underscores the need for awareness and use of effective PPE practices.

## 3 IMPLICATIONS FOR ORAL HEALTH‐CARE PROVIDERS

Health‐care workers such as physicians, nurses, respiratory therapists, dentists, oral health‐care specialists, speech pathologists, ophthalmologists, and otolaryngologists are at an elevated risk of exposure to COVID‐19. Oral health‐care providers (OHCP) are at a high risk in particular for nosocomial transmission of respiratory infectious diseases owing to their proximity to the nasopharynx and oral cavity of patients. The general consensus in dental medicine is that the greatest threat of airborne infection is from aerosols (particles smaller than 50 μm in diameter) due to their ability to stay suspended in the air and contaminate the mucous membranes of the mouth and respiratory passages.^28^, ^29^ Fine aerosols of usually 0.5 to 10 μm in diameter have an even higher potential for transmitting infections. The practice of dental treatment involves the use of surgical and dental equipment, such as aerosol‐generating ultrasonic scalers, air‐water syringes, and handpieces. These instruments create a visible spray of water droplets, salivary spatter, debris, blood, and microorganisms and have the potential to spread nosocomial infections such as tuberculosis and SARS in the exam rooms.^28^, ^29^, ^30^

Currently there are no data available to assess the risk of transmission of SARS‐CoV‐2 in the office settings of dental and specialty practices; however, the Occupational Safety and Health Administration (OSHA) has categorized oral health‐care providers under the “very high exposure risk” category for SARS‐CoV‐2.^31^ OSHA has also recommended the use of powered air‐purifying respirators (PAPRs) or supplied air respirators (SARs) for procedures involving aerosol generation. The Centers for Disease Control and Prevention (CDC) has laid out interim infection control guidance for dental settings,^14^ which includes postponement of all elective procedures, surgeries, and non‐urgent visits. These guidelines advise the use of highest level of PPE available, such as a gown, gloves, eye protective gear (goggles or face shields that cover the front and sides of the face), and N95 or higher‐level respirator during emergency procedures.

The CDC and the American Dental Association (ADA) have recommended that all dentists and oral specialists keep their offices closed and postpone all elective procedures except for urgent and emergency care particularly those in hospital‐based practices, although new guidelines are expected for non‐urgent care.^32^ They have put forth interim guidelines for triaging patients for emergency procedures. The ADA has highlighted special considerations for clinical procedures, such as the use of extraoral radiographs, including panoramic radiographs and cone beam CT over intraoral radiographs, minimizing the use of aerosol‐generating instruments and prioritizing the use of hand instruments, using rubber dams and high‐volume saliva evacuators, and placing resorbable sutures to eliminate the need for follow‐up appointments.^32^ Likewise, the American Association of Oral and Maxillofacial Surgeons (AAOMS) has recommended that non‐aerosol generating procedures and emergent treatments such as antibiotic therapy should be handled in a manner that is as minimally invasive as possible, with the use of adequate PPE.^33^ Previous studies have investigated the virucidal efficacy of pre‐procedural mouthrinse such as 0.23% povidone iodine and 1% hydrogen peroxide, and found them to be highly effective against viruses, including SARS‐CoV.^34^, ^35^ Currently, there are no clinical studies supporting the use of such agents against SARS‐CoV‐2. However, due to its vulnerability to oxidation, topical mouthrinse containing oxidative agents such as povidone iodine may be effective in reducing the salivary viral load of SARS‐CoV‐2.^30^

Special considerations are needed for oral and maxillofacial specialists working in an oncology practice. Initial screening of patients should be conducted via telemedicine consults, and short‐term deferment should be considered. Patients who report a recent travel history to any of the designated COVID‐19 hotspots, and the presence of any symptoms of respiratory illness should be instructed to self‐quarantine for 14 days and report to their physician for COVID‐19 testing. Patients with cancer are considered as highly vulnerable to COVID‐19 due to their immunocompromised status. The MD Anderson head and neck surgery consortium has devised guidelines for triaging patients based on site‐specific head and neck cancers.^36^ All elective oral surgical procedures should be deferred; however, procedures such as planned dental extractions, surgery for patients with early and intermediate malignant disease, and fabrication of oral stents for radiation therapy are to continue. The management of these patients requires a needs assessment on a case‐by‐case basis with patient's primary oncologist, and the interdisciplinary team; and more in depth considerations are beyond the scope and purpose of this report.

Due to the disruption of all major supply chains and increasing global demands, there is a critical shortage of PPE for health‐care personnel. Such an unprecedented situation demands for innovative ideas to address these concerns. Many academic institutions, researchers, and private organizations have come up with creative solutions such as open‐source designs for 3D‐printed respirators and face shields.^37^, ^38^ Customized face shields allow for a more secure fit of the headband, and longer shields are suitable for protection from splatter during dental and surgical procedures.

COVID‐19 is now a widespread and constant presence in our community. As we adapt to this rapidly evolving situation and make adjustments based on new information, revised guidelines from the regulatory authorities will be critical in ensuring the safe reopening of oral health‐care operations. Development and implementation of a rapid COVID‐19 diagnostic test at the point‐of‐care will be vital in safeguarding the health of both patients and OHCP, and minimizing the burden of disease in our community. The American Medical Association (AMA) as of May 1, 2020 stated “as public health experts determine that it is safe to see patients and stay‐at‐home restrictions are relaxed, physician practices should strategically plan when and how best to reopen.” In parallel, the Centers for Medicare and Medicaid Services (CMS) published a Phase 1 guide for reopening facilities to provide non‐emergent, non‐COVID care. The AMA and CMS guidance include pre‐visit screening template and checklists of criteria for reopening private and hospital‐based practices.^39^, ^40^

## 4 CONCLUSIONS

Saliva may be a viable alternative to nasopharyngeal specimen for COVID‐19 testing. Further studies are needed to investigate the efficacy, feasibility and scalability of using salivary specimens for SARS‐CoV‐2 detection and surveillance on a nationwide basis. It is imperative that OHCP stay abreast of the latest developments surrounding the pandemic, follow guidelines from the CDC, ADA, the federal, and state governments, and make informed decisions regarding clinical care. Telemedicine efforts can be an excellent adjunct in triaging patients and determining urgency of need.

## References

[1] Zhu N , Zhang D , Wang W , et al. A novel coronavirus from patients with pneumonia in China, 2019. N Engl J Med. 2020;382(8):727‐733. 31978945 10.1056/NEJMoa2001017 PMC7092803

[2] World Health Organization (WHO). Pneumonia of unknown cause—China. 01/05/2020; https://www.who.int/csr/don/05-january-2020-pneumonia-of-unkown-cause-china/en/. Accessed April 15, 2020.

[3] Lu R , Zhao X , Li J , et al. Genomic characterisation and epidemiology of 2019 novel coronavirus: implications for virus origins and receptor binding. Lancet (London, England). 2020;395(10224):565‐574. 10.1016/S0140-6736(20)30251-8 PMC7159086 32007145

[4] Baker SC , Baric RS . The species Severe acute respiratory syndrome‐related coronavirus: classifying 2019‐nCoV and naming it SARS‐CoV‐2. Nat Microbiol. 2020;5(4):536‐544. 10.1038/s41564-020-0695-z. 32123347 PMC7095448

[5] WHO Director‐General's remarks at the media briefing on 2019‐nCoV on February 11, 2020 [press release].

[6] WHO Director‐General's opening remarks at the media briefing on COVID‐19—March 11, 2020 [press release].

[7] Dong E , Du H , Gardner L . An interactive web‐based dashboard to track COVID‐19 in real time. Lancet Infect Dis. 2020;20:533‐534. 32087114 10.1016/S1473-3099(20)30120-1 PMC7159018

[8] World Health Organization (WHO) . Coronavirus Disease 2019 (COVID‐19) Situation Report—120. Geneva, Switzerland: WHO; 2020.

[9] Zhou P , Yang XL , Wang XG , et al. A pneumonia outbreak associated with a new coronavirus of probable bat origin. Nature. 2020;579(7798):270‐273. 32015507 10.1038/s41586-020-2012-7 PMC7095418

[10] Huang C , Wang Y , Li X , et al. Clinical features of patients infected with 2019 novel coronavirus in Wuhan, China. Lancet (London, England). 2020;395(10223):497‐506. 10.1016/S0140-6736(20)30183-5 PMC7159299 31986264

[11] Yan CH , Faraji F , Prajapati DP , Boone CE , DeConde AS . Association of chemosensory dysfunction and Covid‐19 in patients presenting with influenza‐like symptoms. Int Forum Allerg Rhinol. 2020. 10.1002/alr.22579. PMC7262089 32279441

[12] Wang D , Hu B , Hu C , et al. Clinical characteristics of 138 hospitalized patients with 2019 novel coronavirus‐infected pneumonia in Wuhan, China. JAMA; 2020;323(11):1061‐1069. 10.1001/jama.2020.1585. 32031570 PMC7042881

[13] Zu ZY , Jiang MD , Xu PP , et al. Coronavirus disease 2019 (COVID‐19): a perspective from China. Radiology. 2020;200490. 10.1148/radiol.2020200490. PMC7233368 32083985

[14] Centers for Disease Control and Prevention (CDC) . Interim infection prevention and control guidance for dental settings during the COVID‐19 response. 2020; https://www.cdc.gov/coronavirus/2019‐ncov/hcp/dental‐settings.html. Accessed April 15, 2020.

[15] van Doremalen N , Bushmaker T , Morris DH , et al. Aerosol and surface stability of SARS‐CoV‐2 as compared with SARS‐CoV‐1. N Engl J Med. 2020;382(16):1564‐1567. 32182409 10.1056/NEJMc2004973 PMC7121658

[16] Sabino‐Silva R , Jardim ACG , Siqueira WL . Coronavirus COVID‐19 impacts to dentistry and potential salivary diagnosis. Clin Oral Investig. 2020;24(4):1619‐1621. 10.1007/s00784-020-03248-x PMC7088419 32078048

[17] Hoffmann M , Kleine‐Weber H , Schroeder S , et al. SARS‐CoV‐2 cell entry depends on ACE2 and TMPRSS2 and is blocked by a clinically proven protease inhibitor. Cell. 2020;181:271‐280. 32142651 10.1016/j.cell.2020.02.052 PMC7102627

[18] Xu X , Chen P , Wang J , et al. Evolution of the novel coronavirus from the ongoing Wuhan outbreak and modeling of its spike protein for risk of human transmission. Sci China Life Sci. 2020;63(3):457‐460. 32009228 10.1007/s11427-020-1637-5 PMC7089049

[19] Hamming I , Timens W , Bulthuis ML , Lely AT , Navis G , van Goor H . Tissue distribution of ACE2 protein, the functional receptor for SARS coronavirus. A first step in understanding SARS pathogenesis. J Pathol. 2004;203(2):631‐637. 15141377 10.1002/path.1570 PMC7167720

[20] Zou X , Chen K , Zou J , Han P , Hao J , Han Z . Single‐cell RNA‐seq data analysis on the receptor ACE2 expression reveals the potential risk of different human organs vulnerable to 2019‐nCoV infection. Front Med. 2020;14:185‐192. 32170560 10.1007/s11684-020-0754-0 PMC7088738

[21] Xu H , Zhong L , Deng J , et al. High expression of ACE2 receptor of 2019‐nCoV on the epithelial cells of oral mucosa. Int J Oral Sci. 2020;12(1):8. 32094336 10.1038/s41368-020-0074-x PMC7039956

[22] To KKW , Yip CCY , Lai CYW , et al. Saliva as a diagnostic specimen for testing respiratory virus by a point‐of‐care molecular assay: a diagnostic validity study. Clin Microbiol Infect. 2019;25(3):372‐378. 29906597 10.1016/j.cmi.2018.06.009

[23] To KK , Tsang OT , Chik‐Yan Yip C , et al. Consistent detection of 2019 novel coronavirus in saliva. Clinical Infectious Diseases: An Official Publication of the Infectious Diseases Society of America. New York, NY: Oxford University Press; 2020. 10.1093/cid/ciaa149 PMC7108139 32047895

[24] To KK , Tsang OT , Leung WS , et al. Temporal profiles of viral load in posterior oropharyngeal saliva samples and serum antibody responses during infection by SARS‐CoV‐2: an observational cohort study. Lancet Infect Dis. 2020;20(5):565‐574. 32213337 10.1016/S1473-3099(20)30196-1 PMC7158907

[25] Williams E , Bond K , Zhang B , Putland M , Williamson DA . Saliva as a non‐invasive specimen for detection of SARS‐CoV‐2. J Clin Microbiol. 2020. 10.1128/jcm.00776-20. PMC7383524 32317257

[26] Wyllie AL , Fournier J , Casanovas‐Massana A , et al. Saliva is more sensitive for SARS‐CoV‐2 detection in COVID‐19 patients than nasopharyngeal swabs. medRxiv. 2020. 10.1101/2020.04.16.20067835.

[27] U.S. Food & Drug Administration (FDA) . Rutgers Clinical Genomics Laboratory TaqPath SARS‐CoV‐2 assay accelerated emergency use authorization (EUA) summary. https://www.fda.gov/media/136875/download. Accessed April 14, 2020.

[28] Harrel SK , Molinari J . Aerosols and splatter in dentistry: a brief review of the literature and infection control implications. J Am Dent Assoc. 2004;135(4):429‐437. 15127864 10.14219/jada.archive.2004.0207 PMC7093851

[29] Bentley CD , Burkhart NW , Crawford JJ . Evaluating spatter and aerosol contamination during dental procedures. J Am Dent Assoc. 1994;125(5):579‐584. 8195499 10.14219/jada.archive.1994.0093

[30] Peng X , Xu X , Li Y , Cheng L , Zhou X , Ren B . Transmission routes of 2019‐nCoV and controls in dental practice. Int J Oral Sci. 2020;12(1):9. 32127517 10.1038/s41368-020-0075-9 PMC7054527

[31] Occupational Safety and Health Administration (OSHA) . Guidance on preparing workplaces for COVID‐19. https://www.osha.gov/Publications/OSHA3990.pdf. Accessed April 15, 2020.

[32] American Dental Association (ADA) . ADA Interim Guidance for Minimizing Risk of COVID‐19 Transmission. Chicago, IL: American Dental Association; 2020; https://www.ada.org/~/media/CPS/Files/COVID/ADA_COVID_Int_Guidance_Treat_Pts.pdf?utm_source=adaorg&utm_medium=covid‐statement‐200401&utm_content=cv‐pm‐ebd‐interim‐response&utm_campaign=covid‐19. Accessed April 14, 2020.

[33] American Association of Oral and Maxillofacial Surgeons (AAOMS). Rosemont, IL: American Association of Oral and Maxillofacial Surgeons; 2020. COVID‐19 guidance FAQs. https://www.aaoms.org/practice-resources/covid-19-guidance-faqs. Accessed April 15, 2020.

[34] Eggers M , Koburger‐Janssen T , Eickmann M , Zorn J . In vitro bactericidal and Virucidal efficacy of Povidone‐iodine gargle/mouthwash against respiratory and Oral tract pathogens. Infect Dis Ther. 2018;7(2):249‐259. 29633177 10.1007/s40121-018-0200-7 PMC5986684

[35] Kariwa H , Fujii N , Takashima I . Inactivation of SARS coronavirus by means of povidone‐iodine, physical conditions, and chemical reagents. Japan J Vet Res. 2004;52(3):105‐112. 15631008

[36] Maniakas A , Jozaghi Y , Zafereo ME , et al. Head and neck surgical oncology in the time of a pandemic: subsite‐specific triage guidelines during the COVID‐19 pandemic. Head Neck. 2020;42:1194‐1201. 32342541 10.1002/hed.26206 PMC7267348

[37] Swennen GRJ , Pottel L , Haers PE . Custom‐made 3D‐printed face masks in case of pandemic crisis situations with a lack of commercially available FFP2/3 masks. Int J Oral Maxillofac Surg. 2020;49:673‐677. 32265088 10.1016/j.ijom.2020.03.015 PMC7132499

[38] Prusa Research . 3D Printed Face Shields for Medics and Professionals. Prague, Czech Republic: Prusa Research; 2020; https://www.prusa3d.com/covid19/. Accessed April 15, 2020.

[39] American Medical Association (AMA) . COVID‐19: A physician practice guide to reopening. 2020. https://www.ama‐assn.org/system/files/2020‐05/physican‐guide‐reopening‐practices‐covid‐19.pdf.

[40] Centers for Medicare & Medicaid Services (CMS). Baltimore, MD: U.S. Centers for Medicare & Medicaid Services; Re‐opening facilities to provide non‐emergent non‐COVID‐19 healthcare: Phase 1. 2020; https://www.cms.gov/files/document/covid‐flexibility‐reopen‐essential‐non‐covid‐services.pdf.

